# GABA in the suprachiasmatic nucleus refines circadian output rhythms in mice

**DOI:** 10.1038/s42003-019-0483-6

**Published:** 2019-06-21

**Authors:** Daisuke Ono, Ken-ichi Honma, Yuchio Yanagawa, Akihiro Yamanaka, Sato Honma

**Affiliations:** 1 Department of Neuroscience II, Research Institute of Environmental Medicine, Nagoya University, Furo-cho, Chikusa-ku, Nagoya, 464-8601 Japan; 2 Department of Neural Regulation, Nagoya University Graduate School of Medicine, Nagoya, 466-8550 Japan; 3 Research and Education Center for Brain Science, Hokkaido University Graduate School of Medicine, Sapporo, 060-8638 Japan; 4 Department of Genetic and Behavioral Neuroscience, Gunma University Graduate School of Medicine, Maebashi, Gunma 371-8511 Japan

**Keywords:** Neurophysiology, Circadian regulation

## Abstract

In mammals, the circadian rhythms are regulated by the central clock located in the hypothalamic suprachiasmatic nucleus (SCN), which is composed of heterogeneous neurons with various neurotransmitters. Among them an inhibitory neurotransmitter, γ-Amino-Butyric-Acid (GABA), is expressed in almost all SCN neurons, however, its role in the circadian physiology is still unclear. Here, we show that the SCN of fetal mice lacking vesicular GABA transporter (VGAT^−/−^) or GABA synthesizing enzyme, glutamate decarboxylase (GAD65^−/−^/67^−/−^), shows burst firings associated with large Ca^2+^ spikes throughout 24 hours, which spread over the entire SCN slice in synchrony. By contrast, circadian PER2 rhythms in VGAT^−/−^ and GAD65^−/−^/67^−/−^ SCN remain intact. SCN-specific VGAT deletion in adult mice dampens circadian behavior rhythm. These findings indicate that GABA in the fetal SCN is necessary for refinement of the circadian firing rhythm and, possibly, for stabilizing the output signals, but not for circadian integration of multiple cellular oscillations.

## Abstract (web summary)

Daisuke Ono et al. show that neurons in the suprachiasmatic nucleus from mice lacking VGAT or GAD65/67 exhibit burst firing patterns. These bursts are associated with large calcium spikes and did not affect circadian PER2 rhythms, suggesting an important role for hypothalamic GABA in refinement of circadian output rhythms in mice.

## Introduction

The temporal order of physiology and behavior in mammals is controlled by the master circadian clock located in the suprachiasmatic nucleus (SCN). The SCN generates the endogenous circadian oscillation which entrains to a day–night alternation and synchronizes the peripheral circadian rhythms in a variety of tissues^1^. According to a prevailing hypothesis, the cellular circadian rhythm is generated by an auto-regulatory negative feedback loop involving the clock gene families, *Per*(s), *Cry*(s), *Bmal*(s), and *Clock*, and their protein products^2^. Circadian rhythms of individual SCN neurons are diverse and unstable in period and amplitude, but they are integrated by the neural network to show a coherent circadian rhythm in the output signals such as spontaneous firing^3–6^.

The SCN consists of about 20,000 neurons^7^ and contains a number of neuropeptides and neurotransmitters which play important roles in the circadian organization^8–12^. Among them, γ-aminobutyric acid (GABA) is expressed in almost all SCN neurons^13,14^. GABA is likely involved in the coupling of cellular circadian rhythms^12,15^ or of the regional pacemakers^16,17^ and in the entrainment^15,18^. On the other hand, the mechanism of GABA action in the SCN is still a matter of debate. Controversy still exists on whether GABA is an excitatory or inhibitory neurotransmitter^18–20^ and whether it acts as a synchronizer or a destabilizer of the cellular rhythms^15,21,22^. Previously, we demonstrated that bicuculline, a GABA_A_ receptor antagonist, increased the firing rate of ca. 84% of neurons examined and decreased that of ca. 16% in the dispersed SCN cell culture of neonatal rats, indicating that GABA action is primarily inhibitory but excitatory in some neurons^12^. These controversies are due in part to differences in pharmacological tools and measuring approaches.

The SCN neuronal network is important for the expression of coherent circadian rhythms for the output signals from the SCN^23–25^. In the dispersed SCN cell culture of neonatal rats, circadian rhythms were synchronized in neuron pairs with functional synaptic communications and were not detected in any of the pairs lacking synaptic communication^12^. The firing rate of spontaneous discharge in the cultured SCN on the multi-electrode array dish (MED) ranged typically from 0–1 Hz at the subjective night to 6–10 Hz at the subjective day^6,26^.

GABA is synthesized by a rate-limiting enzyme, glutamate decarboxylase (GAD), and is accumulated in the synaptic vesicles by the vesicular GABA transporter (VGAT)^27^. Two isoforms of GAD, GAD65 that primarily locates in axon terminals and GAD67 distributed throughout the cells, are encoded by separate genes^28^. There was virtually no GABA content in the GAD65/GAD67 knockout mouse brains^29^ and very little GABAergic inhibitory postsynaptic currents were detected in neuronal cultures from VGAT knockout striatum^30^.

In the present study, we examined GABA signaling in the SCN using mice lacking VGAT (VGAT^−/−^) or GAD65 and GAD67 (GAD65^−/−^/67^−/−^). We simultaneously measured the circadian rhythms with a bioluminescence reporter for the clock gene product PER2 (PER2::LUC), spontaneous firing and intracellular calcium (Ca^2+^) level for several circadian cycles in the cultured SCN slices of perinatal mice. Here we demonstrate that the SCN lacking GABA exhibits burst firings of 35–80 Hz at 2–3 min intervals throughout 24 h. A burst firing was associated with an abrupt increase in intracellular Ca^2+^, which was synchronous throughout the entire SCN slice. By contrast, the circadian PER2 rhythm was essentially kept intact. We also found that SCN-specific VGAT depletion in the adult mice showed a circadian behavior rhythm with a decreased amplitude and a large cycle-to-cycle variation. GABA in the SCN may refine the circadian firing rhythms which are important as output signals.

## Results

### Disruption of GABA signaling induced burst firing in the fetal SCN slice

We examined the effect of GABA deficiency on the circadian rhythms of spontaneous firing, intracellular Ca^2+^ level, and circadian PER2 rhythm in the fetal SCN cultured on MED. Since the VGAT^−/−^ mice do not survive after birth^31^, we obtained VGAT^−/−^ SCN slices at the embryonic day 19 or 20 together with the wild type (WT) and VGAT^+/−^ littermates from VGAT^+/−^ females crossed with VGAT^+/−^ males.

Multiunit activity in the SCN slice was measured continuously for more than 5 circadian cycles and spontaneous discharges with signal to noise ratio (*S*/*N*) > 2 were collected from each electrode (Supplementary Fig. 1). The frequency of burst firings (Hz) was evaluated for 10 min records in 100 ms bins (6000 bins) at CT8 when the highest neuronal activity was observed in both VGAT^−/−^ and WT mice (Supplementary Fig. 2) and the relative abundance of spike expressed as the ratio (%) of bin numbers to the total was plotted against the frequency (Supplementary Fig. 3a). The bin ratio showed a marked decrease around 10 Hz and never exceeded 35 Hz in the WT SCN, whereas more than 3% of the total number of bins exceeded 35 Hz in the VGAT^−/−^ SCN (Supplementary Fig. 3b). Thus, the firing rate over 35 Hz was regarded as the burst firing in this study. The burst firing abruptly appeared for a few seconds and repeated at 2–3 min intervals (Supplementary Fig. 1). Representative patterns of burst firing in other SCN slices are also illustrated in Supplementary Fig. 2.

**Fig. 1:**
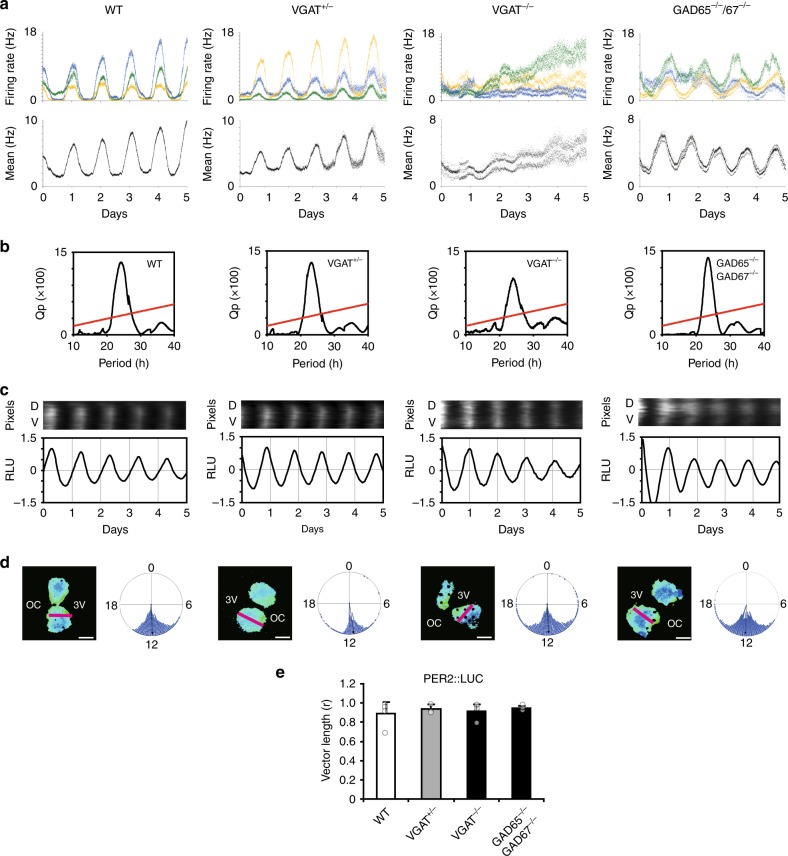
Circadian firing and PER2::LUC rhythms in the VGAT^−/−^ and GAD65^−/−^/67^−/−^ fetal SCN. **a** Spontaneous firing rate (Hz) plotted in 1 min bins for 5 circadian cycles from three electrodes (upper) in a single SCN slice and the average of all electrodes (*n* = 15, 13, 13, and 13 for WT, VGAT^+/−^, VGAT^−/−^, and GAD65^−/−^/67^−/−^) (lower) located on the SCN region of a cultured slice from WT (far left), VGAT^+/−^ (left), VGAT^−/−^ (right), and GAD65^−/−^/67^−/−^ (far right) mice. The different colors indicate the firing rhythms from different electrodes on MED. **b** The Chi-square periodogram of spontaneous firing rhythm in the SCN illustrated in the above panel. The oblique red line in the periodogram indicates the significance level (*p* < 0.01). **c** Circadian rhythms of PER2::LUC at the pixel level illustrated in a line scan (1 pixel width) from the dorsal (D) to the ventral (V) region (upper) and on whole tissue level (lower) of the same SCN as indicated in (**a**). The site of the line scan is shown as a red line in the corresponding acrophase map in (**d**). **d** Acrophase map of PER2::LUC rhythms (left) and phase distribution of each pixel in a form of Rayleigh plot (right). Black spots in the phase map of WT, VGAT^−/−^, and GAD65^−/−^/67^−/−^ SCN indicate electrodes. Scale bars represent 200 µm. OC: optic chiasm, 3V: third ventricle. **e** Mean vector length, *r*, calculated from the phase map. The number of SCN used are *n* = 5, 4, 6, and 4 for WT, VGAT^+/−^, VGAT^−/−^, and GAD65^−/−^/67^−/−^, respectively. Phase distribution was not different among the four genotypes (*p* = 0.728, one-way ANOVA)

The mean firing rate was calculated at every 1 min from all electrodes located at the SCN region (9–19 electrodes/SCN, Supplementary Figs. 1 and 2). The firing rate was typically in the range of 2–18 Hz at the circadian peak in the WT SCN (Fig. 1a, b). By contrast, the circadian firing rhythms in the VGAT^−/−^ and GAD65^−/−^/67^−/−^ SCN were characterized by two distinct bands of a lower and higher firing rate throughout the circadian phase (Fig. 1a from the VGAT^−/−^ and GAD65/67^−/−^ SCN). The band of higher firing rates represented the bins containing the burst firings (higher than 35 Hz) and the band of lower firing rates represented those without burst firings. The two bands were detected from the start of culture and the difference in the mean frequency (Hz) between the two bands was not changed throughout the course of culture (Fig. 1a, Supplementary Fig. 4a), whereas the firing rate itself steadily increased during this period in the VGAT^−/−^ SCN.

**Fig. 2:**
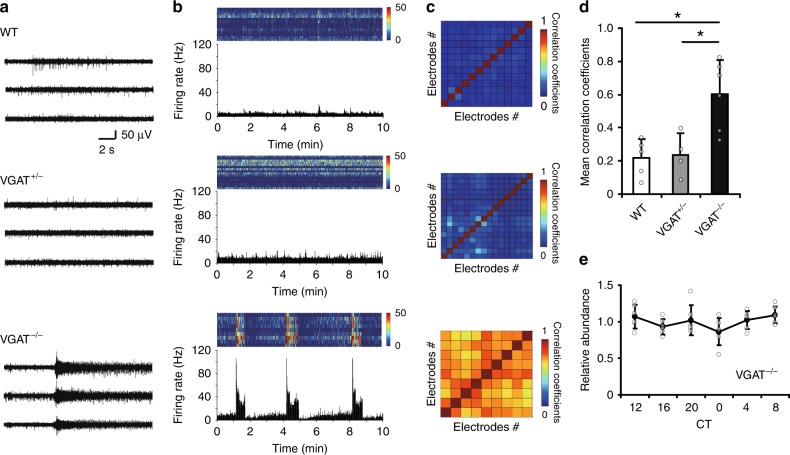
Synchronous burst firings in the entire SCN of fetal VGAT^−/−^ mice. **a** Representative spontaneous firings at each of the three MED electrodes located on a unilateral SCN from WT (upper), VGAT^+/−^ (middle) and VGAT^−/−^ (lower) mouse. **b** Raster plots of firing rates of individual electrodes expressed in pseudocolor (upper) and their mean (lower). The color key of the firing rate (Hz) is indicated in the right margin. Records for 10 min at CT8 are shown for the respective slices, where CT12 is defined as the peak phase of PER2::LUC rhythms. Burst firings were observed only in VGAT^−/−^ SCN. **c** Correlation matrix heat maps showing the correlation coefficient for all pairs of electrodes on the respective SCN slice. Each square indicates an electrode, and a red diagonal line denotes correlations at the same electrode. A high correlation coefficient was detected in all electrodes of VGAT^−/−^ SCN. Data in (**a**–**c**) are obtained from the same SCN slice for each genotype. **d** The mean correlation coefficient of all SCN slices examined (WT, *n* = 5; VGAT^+/−^, *n* = 4; VGAT^−/−^, *n* = 6). The correlation coefficient of VGAT^−/−^ SCN was significantly larger than that of the other two genotypes (*p < 0.05, versus WT or VGAT^+/−^, one-way ANOVA with a post-hoc Tukey–Kramer test). **e** A lack of phase dependency in the number of burst firing (mean ± SD, *n* = 6) for the VGAT^−/−^ SCN. The number of bins containing burst firings (>35 Hz) was counted for 10 min at every 4 h, and expressed as a relative value to the daily mean (*p* = 0.198, one-way repeated measure ANOVA)

Burst firings in the VGAT^−/−^ SCN were detected in all electrodes in synchrony (Fig. 2a, b) throughout the entire SCN slice and strongly correlated in time with each other as shown in the heat map of correlation coefficients (Fig. 2c) which was significantly high in the VGAT^−/−^ SCN (Fig. 2d). Importantly, the occurrence of burst firing in the VGAT^−/−^ SCN was not dependent on the circadian phase (Fig. 2e, Supplementary Fig. 5). The firing rate and shape of burst varied among the SCN slices but was indistinguishable among the electrodes in the same SCN (Supplementary Fig. 2).

Despite the superposition of burst firing, Chi-square periodogram revealed significant (*p* < 0.01) circadian firing rhythms in the VGAT^−/−^ and GAD65^−/−^/67^−/−^ SCN (Fig. 1b). Their circadian periods (VGAT^−/−^, 23.63 ± 0.55 h; GAD65^−/−^/67^−/−^, 23.79 ± 0.44 h) were comparable to those of the WT (23.86 ± 0.42 h) and VGAT^+/−^ (23.35 ± 0.18 h).

### Circadian PER2 rhythms were kept intact in the VGAT^−/−^ and GAD65^−/−^/67^−/−^ SCN

To understand the effects of GABA deficiency on the SCN molecular clock, we examined the circadian PER2 rhythms in SCN slices from VGAT^−/−^ and GAD65^−/−^/67^−/−^ mice carrying a bioluminescence reporter for the clock gene product PER2 (PER2::LUC)^32^. Bioluminescence emitted from the PER2 reporter was monitored with an electron-multiplying charge-coupled device (EM-CCD) camera together with spontaneous firing in the SCN.

PER2::LUC rhythms in the VGAT^−/−^ and GAD65^−/−^/67^−/−^ SCN were as robust as those in WT or VGAT^+/−^ littermates (Fig. 1d, Supplementary Fig. 6a). Cosine curve fitting was applied to all pixels (4.3 μm × 4.3 μm) to obtain the acrophase (fitted circadian peak phase) (Fig. 1d). Acrophase maps (Fig. 1d, left) and distribution of acrophases by Rayleigh plot (Fig. 1d, right) indicated that the PER2::LUC rhythm of each pixel was synchronized in the entire VGAT^−/−^ SCN slice to a similar extent to those in the WT SCN. In addition, the distribution of the acrophase in terms of the mean length of vector (*r*) was not different among genotypes (Fig. 1e). The same results were obtained in the simultaneously recorded PER2::LUC and spontaneous firing of the GAD65^−/−^/67^−/−^ SCN (Fig. 1a–e). The variability of the circadian period in terms of SD was not significantly different among genotypes.

The PER2::LUC rhythms of four genotypes were also recorded by photo multiplier tube (PMT). The circadian period, the standardized amplitude in the first circadian cycle and the damping ratio during the first six cycles were not significantly different among them (Supplementary Fig. 6), which were consistent with those of circadian firing rhythm measured on MED (Fig. 1). These results indicate that GABA is neither necessary for the generation nor the synchronization of the circadian PER2::LUC rhythm in SCN cells.

### Rescue and mimic of burst firings with pharmacological manipulation of GABA signaling

**Fig. 3:**
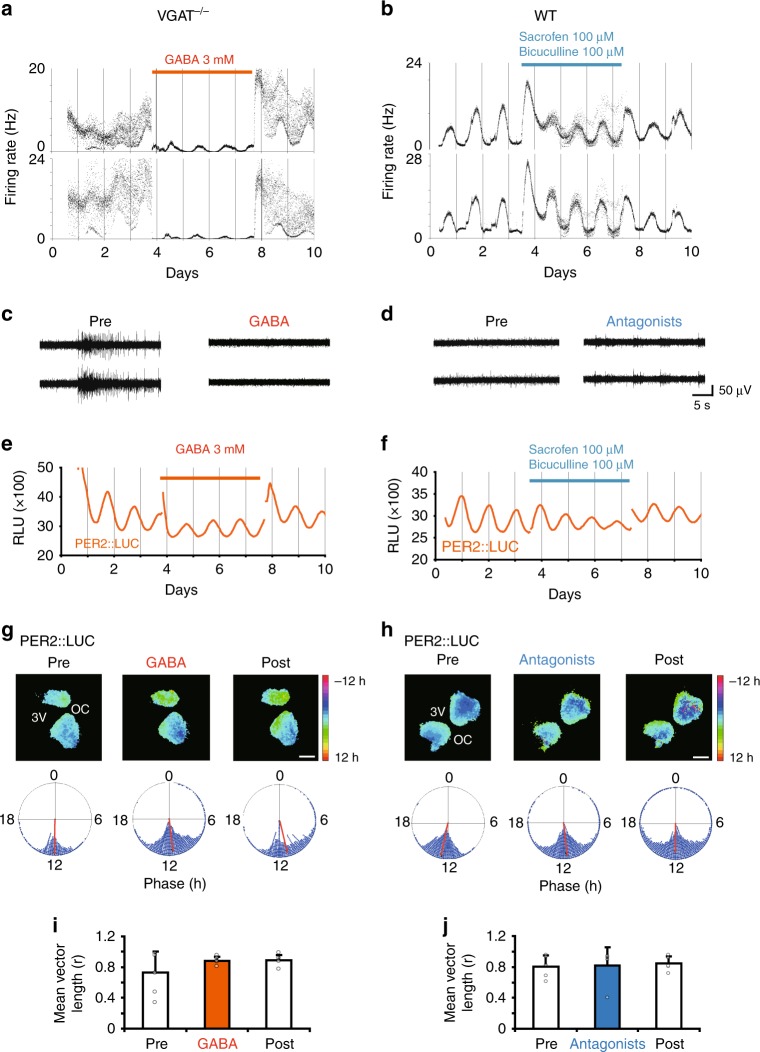
Effects of pharmacological manipulation on spontaneous firings and circadian PER2 rhythms in the fetal SCN. **a** Effects of GABA application (3 mM) and **b** GABA receptor antagonists (bicuculline and sacrofen, final concentration 100 μM) on the frequency of spontaneous firing in 1 min bins. Representative results are illustrated from two electrodes in a VGAT^−/−^ and WT SCN slice, respectively. A red horizontal bar indicates the period of GABA application and a blue horizontal bar, the period of GABA receptor antagonists. Drug treatment was done from the 3rd to 7th culture day (ca. 96 h). **c**, **d** Representative neuronal activity before (pre) and during treatment (GABA or receptor antagonists) on the same electrodes as shown in (**a**) and (**b**). **e**, **f** Circadian PER2::LUC rhythms from the entire SCN area before, during and after the treatment with GABA and GABA receptor antagonists in the VGAT^−/−^ (**e**) and WT (**f**) SCN. See also the legend for (**a**) and (**b**). The PER2 rhythms were monitored simultaneously with spontaneous firings as shown in (**a**) and (**b**). **g**, **h** Acrophase-maps (upper) and -distribution (lower) of circadian PER2::LUC rhythm in VGAT^−/−^ (**g**) and WT (**h**) SCN before (pre), during and after (post) pharmacological treatment. The acrophase at pixel level is normalized relative to the mean phase of the whole slice and expressed in pseudocolor. A color key indicates the relative circadian phase from the slice mean. 3V: the third ventricle, OC: optic chiasm. A scale bar: 200 μm. Acrophase distribution in the phase map was expressed in a Rayleigh circle. A red line in the circle indicates a distribution vector. **i**, **j** Mean vector length in VGAT^−/−^ (*n* = 6) and WT (*n* = 5) SCN treated with GABA and GABA receptor antagonists, respectively. No significant difference was detected in the length of vector before (pre) during (antagonists or GABA) and after (post) pharmacological treatment. (VGAT^−/−^, *p* = 0.272; WT, *p* = 0.921, one-way repeated measures ANOVA)

VGAT or GAD65/67 deficiency induced burst firings of high frequency in the SCN slice, which could be due to a lack of tonic release of GABA in the SCN. In order to test this possibility, we applied GABA in the culture medium for the VGAT^−/−^ SCN slice to see whether the noisy circadian rhythm with superimposed burst firings was rescued or not. As expected, burst firings in the VGAT^−/−^ SCN were abolished by GABA application, the effective dose of which was as little as 3 mM (Fig. 3a, c, Supplementary Figs. 7 and 8a, b). Lower doses failed to reduce the burst firing (Supplementary Fig. 7). Importantly, even during GABA application, nearly a half of the electrodes examined (44%, 36/82, *n* = 5 slices) showed significant circadian rhythms in neuronal activity without burst firings, but the rest of electrodes did not exhibit any neuronal activity (56%, 46/82, *n* = 5 slices). Pixel level analysis of PER2::LUC images revealed that GABA application did not affect the circadian rhythms or distribution of the acrophases (Fig. 3e, g, i). These results support the conclusion that burst firings are ascribed to a lack of GABA signaling.

**Fig. 4:**
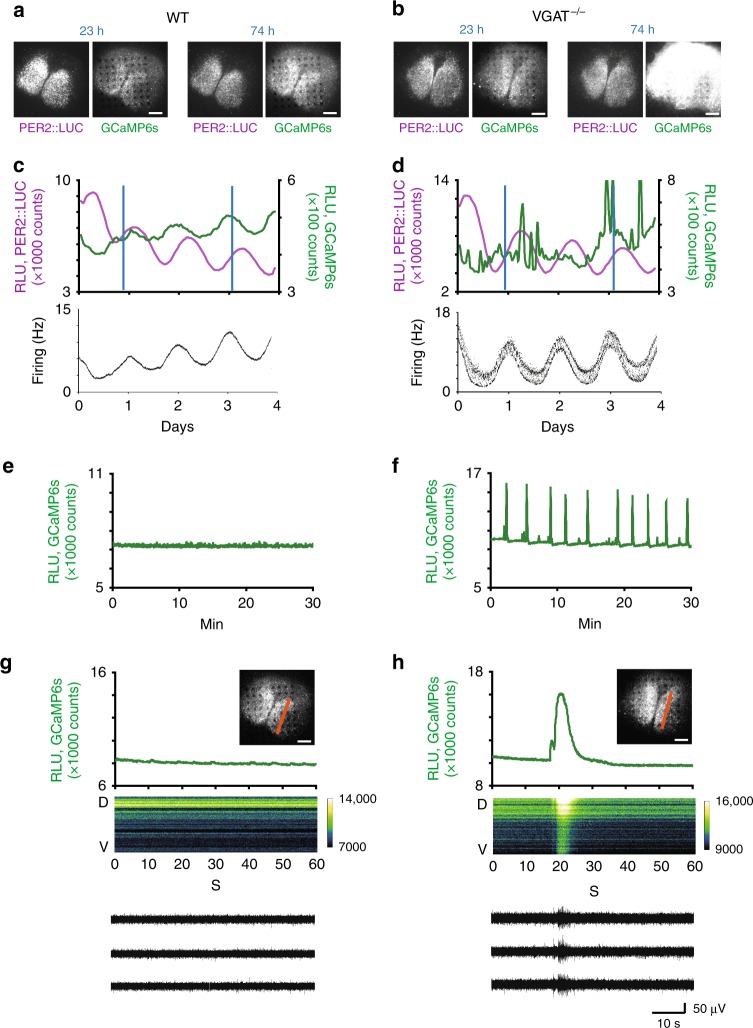
Intracellular Ca^2+^ monitored simultaneously with PER2::LUC bioluminescence and spontaneous firing in the fetal SCN slice on MED. **a**, **b** Intracellular Ca^2+^ levels and PER2::LUC bioluminescence measured every 1 h from a WT (**a**) and VGAT^−/−^ (**b**) SCN slice on MED. Measurements were taken at 23 h (left) and 74 h (right) after the start of culture. Black spots in the SCN slice indicate electrodes. **c**, **d** Spontaneous firing was measured every 1 min. PER2::LUC and calcium rhythms were measured at every 1 h and at 3 s, respectively. The VGAT^−/−^ (**d**) but not the WT (**c**) SCN exhibited calcium spikes. **e**, **f** Fluctuation of intracellular Ca^2+^ at 0.33 fps for 30 min. **g**, **h** Ca^2+^ level fluctuation at 10 fps for 60 s with an inserted fluorescence image of an SCN superimposed by a red line (upper), a raster plot of Ca^2+^ fluctuations scanned along the orange line from dorsal to ventral (middle) and spontaneous firings at three electrodes (lower). Ca^2+^ levels are expressed in pseudocolor, the color key of which is shown at the right margin. Calcium spikes were synchronous throughout the entire SCN slice and coincided in time with burst firings in VGAT^−/−^ SCN

To test whether or not suppression of GABA action mimics the burst firings, a cocktail of GABA_A_ and GABA_B_ receptor antagonists, bicuculline and sacrofen, was applied to the WT SCN slice. Upon application of GABA antagonists, the WT SCN showed burst firings similar to those detected in the VGAT^−/−^ SCN (Fig. 3b, d, Supplementary Fig. 8c, d). The firing rate showed two bands which persisted for 4 days. The circadian firing rhythms were still obvious even though superimposed by burst firings. The circadian PER2::LUC rhythm was not affected at all by GABA antagonists (Fig. 3f). The standardized circadian amplitude (from trough to peak divided by peak value) on the cycle immediately before the drug application and that on the second cycle after the application were not significantly different. PER2::LUC images revealed that GABA antagonists did not change the circadian phase distribution on pixel level (Fig. 4h, j). These results support the conclusion that burst firings are due to a lack of GABA signaling.

### VGAT deficiency produced calcium spikes in the fetal SCN

Genetic deficiency of GABA in the SCN modulated circadian rhythms in neural firing, without affecting the circadian molecular oscillation (Fig. 1, Supplementary Fig. 5). Intracellular Ca^2+^ showed circadian rhythms^33,34^ and played an important role in the transmission of input and output signals to and from the cellular circadian oscillation in the SCN^33–36^. To investigate the effects of VGAT deficiency on circadian Ca^2+^ rhythms, hSyn-GCaMP6s, a fluorescent intracellular Ca^2+^ sensor was introduced into the cultured SCN slice carrying a PER2::LUC reporter using adeno-associated virus (AAV). The slice was set on an MED probe for simultaneous measurement of fluorescence (Ca^2+^), bioluminescence (PER2::LUC), and spontaneous firing.

The WT SCN slice showed robust circadian PER2::LUC, intracellular Ca^2+^, and spontaneous firing rhythms (Fig. 4a, c, Supplementary Movie 1) as previously reported^33,37^. Interestingly, intracellular Ca^2+^ in the VGAT^−/−^ SCN occasionally showed a spike-like increase which was superimposed on the circadian Ca^2+^ rhythm, while PER2::LUC exhibited robust circadian rhythms similar to those of the WT SCN (Fig. 4d, Supplementary Movie 2). The circadian firing rhythms showed two distinct bands. Time-lapse Ca^2+^ imaging at 3 s intervals for 30 min revealed the calcium spikes in the VGAT^−/−^ but not in the WT SCN (Fig. 4e, f, Supplementary Movie 3). In order to examine the temporal relationship between the burst firing and the calcium spike, Ca^2+^ imaging was performed with a higher time resolution at 100 ms intervals for 1 min (Fig. 4g, h, Supplementary Movie 4). The calcium spikes occurred synchronously throughout the entire SCN slice for several seconds (Fig. 4d, f, h). The membrane potential also showed burst firings throughout the slice in synchrony with calcium spikes (Fig. 4h). These findings indicate that lack of GABA signaling induces a spike-like increase in intracellular Ca^2+^ throughout the entire SCN slice in synchrony with burst firings.

### VGAT deletion in the SCN attenuates circadian behavioral rhythms

**Fig. 5:**
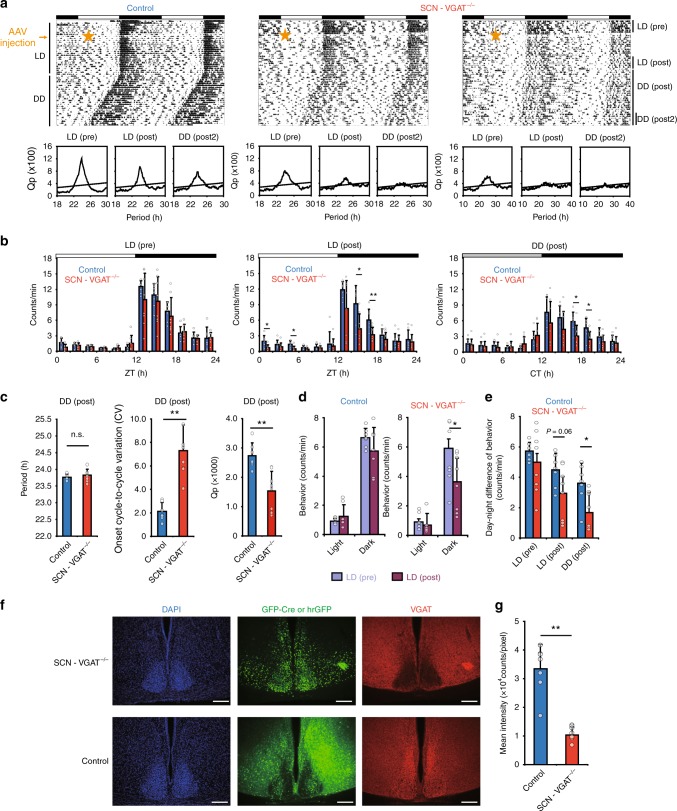
Circadian behavioral rhythms of adult mice with the SCN specifically depleted VGAT. **a** Representative locomotor activity of control (left) and SCN-VGAT^−/−^ (middle and right) mice is double plotted. Chi-square periodogram during LD (pre, 7 days before AAV injection), LD (post, 14–21 days after injection), and DD (post, 21–28 days after exposed to DD) are exhibited under each actogram. AAV was injected on the day indicated by a yellow star. Three weeks after AAV injection, mice were released to DD. **b** Daily profiles of circadian behavioral rhythms under LD (pre), LD (post), and DD (post, 1–28 days after exposed to DD) indicated in (**a**). Control (blue, *n* = 7) and SCN-VGAT^−/−^ (red, *n* = 8) mice were plotted every 2 h. The amount of activity during the subjective night was significantly decreased in the VGAT^−/−^ SCN compared to that of the WT. Two-way repeated measure ANOVA revealed a significant interaction between time of day and genotype in LD (post) and DD (post) (**p < 0.01, *p < 0.05, Two-way repeated measure ANOVA post-hoc *t*-test). Black and white horizontal bars on the top of the actograms indicate the dark and light phase of an LD cycle, respectively. Data are expressed as mean ± SD. **c** Circadian period calculated by a periodogram (left) (*p* = 0.447, Student’s *t*-test), cycle-to-cycle variation of the onset phases (middle) (**p = 0.00014, Welch’s *t*-test), and Qp value obtained from periodogram (right) (**p = 0.0015, Student’s *t*-test) from the WT and VGAT^−/−^ SCN mice were calculated using data from 28 days after DD exposure. **d** The amount of behavioral activity under the light and dark period were compared between LD (pre) and LD (post) (**p = 0.0023, paired *t*-test). **e** Difference in the amount of behavioral activity between the light and dark or the subjective day and night during free-running was exhibited during LD (pre), LD (post), and DD (post) period (*p = 0.016, Student’s *t*-test). **f** Representative pictures of VGAT immunostaining (red), GFP-Cre or hrGFP (green), and DAPI (blue) from WT and VGAT^−/−^ SCN. Scale bars are 200 μm. **g** Mean intensity of VGAT immunostaining in the WT and VGAT^−/−^ SCN was significantly different (**p = 0.00024, Welch’s *t*-test)

To understand the role of GABA in the SCN in the output signal to behavior, mice with an SCN-specific deletion of VGAT were generated by injecting AAV9-hSyn-GFP-Cre into the SCN of VGAT^flox/flox^ mice (SCN-VGAT depleted mice). As control, AAV9-hSyn-hrGFP was injected into the SCN of VGAT^flox/flox^ mice. Three weeks later, mice were transferred from a light–dark (LD) cycle to constant darkness (DD). Circadian behavioral rhythms were persisted in SCN-VGAT depleted mice but the activity was disrupted under DD (Fig. 5a–e). The amount of behavioral activity in the dark period of the LD cycle was reduced in the SCN-VGAT depleted mice (Fig. 5d) and the subjective day–night difference in the activity was also reduced under DD (Fig. 5f). The cycle-to-cycle variability increased and the Qp value in the Chi-square periodogram decreased in the SCN-VGAT depleted mice (Fig. 5c). However, the free-running period was not different between the SCN-VGAT depleted and control mice. Post-hoc immunostaining revealed that VGAT expression was significantly attenuated in the SCN of SCN-VGAT depleted mice compared to the control SCN by ca. 31% (Fig. 5f, g). These results suggest that a lack of GABA function in the SCN deteriorates circadian behavioral rhythms.

## Discussion

We found that burst firings of 35–80 Hz appeared in the cultured SCN slice of fetal mice and obscured the circadian firing rhythm when GABA signaling was genetically or pharmacologically disrupted. Calcium spikes, which synchronized with burst firings, were also observed in GABA-deficient SCN. GABA application rescued the noisy neuronal activity in the VGAT deficient SCN slice. By contrast, disruption of GABA signaling did not affect the PER2 circadian rhythm. Mice with deleted VGAT specifically in the SCN showed damped circadian behavioral rhythms without changes in the free-running period. From these findings, we conclude that GABA refines the circadian firing rhythm in the fetal SCN to make output signals clear.

Burst firings similar to those observed in the VGAT^−/−^ and GAD65^−/−^/67^−/−^ SCN slices were reported in the neurons outside the SCN such as the dopaminergic neurons in the substantia nigra and ventral tegmental area^38,39^. In the present study, burst firings were observed in non-SCN neurons in the cultured SCN slice on MED (Supplementary Fig. 2), indicating that burst firings are not unique to the SCN neurons. Previously, we observed fast synchronous oscillations of the firing activity in the dispersed cell culture of neonatal rat SCN slice on MED, which appeared in synchrony with other neurons^40^. The fast synchronous oscillation occurred at approximately 1–2 min intervals similar to the burst firing in the present study. Recently, Tsuji et al.^41^ demonstrated harmonic burst firings of close to 30 Hz in light-responsive SCN neurons in adult rats. The firing rate of the harmonic bursts was not different between day and night, which is similar to the present study. However, burst firings are not common in the adult SCN neurons^42–44^. Therefore it is a matter of discussion whether the synchronous burst firings throughout the SCN are specific for the neonates or not.

Importantly, GABA application abolished the burst firings in the VGAT^−/−^ SCN slice, and GABA receptor antagonists in the WT SCN induced bursting without affecting the circadian oscillation (Fig. 3). These findings lead us to the hypothesis that the burst firing in the fetal SCN is caused by disinhibition of the neuronal activity through depleting the GABA signaling, which has been observed in other brain structures^45^.

Burst firings spread rapidly over the entire area of fetal SCN slice, without affecting the period and the phase of circadian PER2::LUC rhythm. The findings indicate that GABA is not involved in the neural network which is responsible for the coupling of oscillating neurons to build up coherent circadian rhythms in the SCN, partly consistent with the previous report using the GABA_A_ receptor antagonist^21,22^. In the neural network for the oscillatory coupling, VIP and AVP, and probably other molecules are involved^9,11^.

Previously, GABA receptor antagonists have been reported to block phase shifts by a light pulse, suggesting that GABA is involved in light entrainment^46,47^. The retinohypothalamic tract for light entrainment is not developed yet in the fetal or perinatal SCN^48^. Fetus circadian rhythms are entrained by pregnant dam^49^, which is unlikely disturbed by GABA deficiency, since the first circadian phase in culture of GABA-deficient SCN was not different from that in WT SCN. On the other hand, GABA was reported to produce a phase-dependent phase-shift of the circadian rhythm in vivo^50,51^ and in cultured SCN neurons^15^. Although the resulting phase response curves are different, a dark pulse type in behavioral rhythms versus a light pulse type in dispersed SCN neurons, GABA is possibly involved in the neural system of rhythm entrainment^52^. The present finding was not contradictory to the above hypothesis, because the burst firing due to GABA deficiency covered all circadian phases, which could cancel out the phase-shifting effects of GABA, if any. The present findings support the idea of differential neural networks for the regulation of circadian oscillation in the SCN^21^.

GABA is a major inhibitory transmitter in the central nervous system but also an excitatory transmitter depending on the developmental stage^53^ and on the areas of brain. According to a current agreement^54^, the activation of GABA_A_ receptor opens chloride (Cl^−^) channels on the cell membrane to allow inward flow of Cl^−^ resulting in hyperpolarization (inhibitory action), when the Cl^−^ equilibrium potential level is lower than the resting membrane potential. The opposite (excitatory) is the case, when the Cl^−^ equilibrium potential level is higher than the resting potential. In the SCN, the switch from inhibitory action to excitatory and vice versa was reported to occur depending on the circadian phase and the area in the SCN^18,20^. We demonstrated previously that GABA action was changed alternatively in some synchronous SCN neurons in which GABA was excitatory at the circadian peak and inhibitory at the circadian trough^12^. In the present study, GABA application to the fetal SCN slice of VGAT^−/−^ mice exerted tonic inhibition and GABA receptor antagonists to the WT SCN exerted tonic excitation, both of which did not interfere with the circadian molecular rhythm.

It is not known whether the burst firing occurs in the adult SCN lacking GABA signaling. The perinatal and adult circadian pacemakers differ in several aspects. The SCN circadian system is immature in the perinatal period with respect to light entrainment and oscillatory coupling^11,55^. Before neural pruning which occurs around the postnatal day 2–5^56^, the number of neurons is more abundant but the synaptic connections are less abundant in the perinatal SCN than in the adult^56^. The perinatal SCN entrains not to light cycles but to maternal circadian rhythms^57^. VIP, an important neuropeptide for coherent circadian rhythms in the SCN, shows an endogenous circadian rhythm in the neonatal SCN but not in the adult, indicating that VIP is regulated by the circadian pacemaker more strongly in the perinatal period than in the adulthood^11^. Recently, the neural network for the coupling of cellular circadian oscillators is suggested to be different among the embryonic, neonatal, and adult SCN^11,55,58–61^. However, the developmental change in the GABA system remains to be studied.

The mechanism of burst firing generation has not been elucidated^62^. Burst firings are general phenomena in the central nervous system outside the SCN under physiological conditions^63^. In contrast, the WT SCN slice lacks burst firings. The intact SCN neurons in a dispersed cell culture showed more noisy firing rhythms superimposed by fast oscillation at 1–2 min intervals^40^ than in a slice culture with sinusoidal firing rhythm^3,6,23^. These features have similarities to the burst firings in the SCN slice lacking GABA signaling. Neural communication becomes less potent as the distances among the SCN neurons get larger in dispersed cell cultures^12^. Similar to dispersed neurons, the SCN neural network may attenuate its potency to communicate with other neurons with GABA signaling and becomes noisy. Burst firings could be an intrinsic nature of neurons or neural networks, and GABA in the SCN, at least in the perinatal SCN where the neural networks are immature, erases noises from the circadian signals.

The firing rates in the cultured SCN increased during culture, suggesting an increase in neural connections (Fig. 1a, Supplementary Fig. 4a). The burst firing was detected from the very beginning of SCN culture and the difference in burst and non-burst firings (difference between two bands) did not change throughout the culture period, indicating that changes in neural networks during culture do not contribute to the appearance of burst firings.

The burst firing was associated with an abrupt increase in intracellular Ca^2+^ (Fig. 4h). In presynaptic neurons, GABA release is induced by an influx of Ca^2+^ through voltage-gated ion channels. The firing rate determines the amount of Ca^2+^ influx, altering the intracellular Ca^2+^. On the other hand, in postsynaptic neurons, GABA-induced hyperpolarization (inhibitory action) decreases intracellular Ca^2+^, whereas GABA-induced depolarization (excitatory action) increases Ca^[2+54^. In the SCN, the effects of GABA are complex and could be different depending on co-localized peptides, the areas in the SCN, circadian phases and photoperiods^20,64,65^. It is interesting to note that the circadian PER2::LUC rhythm persisted despite frequent bursts of intracellular Ca^2+^ in the SCN through inhibition of the GABA signaling. The circadian rhythm in intracellular Ca^2+^ received a dual regulation from the neural networks and the core molecular loop. Even when the neural inputs were shut down by the sodium channel blocker Tetrodotoxin, the circadian Ca^2+^ rhythm persisted with a small change in amplitude^33,34,66^. Intracellular Ca^2+^ is suggested to affect the molecular loop for circadian rhythm generation through CaMKll or CREB^1^. The burst-like increase in intracellular Ca^2+^ levels may bypass the routes to the molecular loop.

The SCN-specific reduction of VGAT in adult mice decreased the activity level during the dark period and increased cycle-to-cycle variability of circadian behavioral rhythms. However, the circadian rhythm was kept entrained to LD and the mean free-running period was not different from that of WT mice, partially confirming the previous pharmacological experiments^21,22^. These results were essentially consistent with slice experiments in the fetal SCN, although the suppression of VGAT level was not complete (ca. 69%) in the adult.

Coherent circadian rhythms in neuronal activity are important in the output of the SCN, since the complete isolation of the SCN from other brain structures by Halàsz knife interrupts the transmission of circadian signals to behaviors^67^. GABA plays a critical role in refining the circadian firing rhythm in the fetal SCN. In this respect, the present results were not consistent with a previous study in which GABA_A_ receptor antagonists were reported to decrease the variability of circadian period of PER2::LUC rhythms in cultured SCN, suggesting destabilization of the circadian molecular oscillation by GABA_A_ signaling^22^. The discrepancy could be due to a different approach to GABA signaling; bath application of chemicals into culture medium^22^ and genetic manipulation of the endogenous GABA system in the present study. Furthermore, in the present study we used the fetal SCN, while the previous study monitored the adult SCN. The SCN in hamster^68^ and mice^69^ underwent developmental cell death and about 40% decrease was detected in the first 3 postnatal days^68^. Therefore, GABA may reduce the noisy firings in the SCN before developmental neuronal death occurs.

In conclusion, GABA is necessary for suppressing the burst firing of neuronal activity and abrupt increases of intracellular Ca^2+^ levels but not for the generation and stability of molecular circadian oscillation in the fetal SCN. The GABA network may refine the circadian firing rhythm to ensure noiseless communications with the neurons outside the SCN.

## Methods

### Animals

VGAT^−/−^^70^ and GAD65^−/−^/67^−/−^^71^ mice on the C57BL/6J background and VGAT^flox/flox^ ^70,72^ mice on the 129/SvEv (back-crossed with C57BL/6J mice for at least two generations) were used. They were bred with *mPer2*^*Luc*^ knock-in mice carrying a PER2::LUC fusion reporter^32^. Mice were bred and reared in the animal quarter at Hokkaido University Graduate School of Medicine, where environmental conditions were controlled (lights-on 6:00–18:00 h; light intensity approximately 100 lx at the bottom of cage: humidity 60 ± 10%). Both male and female mice were used in the present study. Experiments were conducted in compliance with the rules and regulations established by the Animal Care and Use Committee under the ethical permission of the Animal Research Committee of Hokkaido University (Approval No. 08-0279), Nagoya University (Approval No. 18257), and Gunma University (Approval Nos. 07-109, 09-047, and 14-006).

### Preparation of fetal SCN slice for monitoring circadian rhythms

Since VGAT^−/−^ and GAD65^−/−^/67^−/−^ die postnatally, heterozygote mice (VGAT^+/−^ or GAD65^+/−^/67^+/−^) were mated to obtain homozygote fetuses (VGAT^−/−^ or GAD65^−/−^/67^−/−^). The fetal SCN were taken under isoflurane anesthesia at the embryonic day 19 or 20 and the fetus genotype was examined later. Coronal brain slices were cut 300 μm with a tissue chopper (Mcllwain) and the SCN was dissected at the mid rostro-caudal region. A paired SCN was cultured on a Millicell-CM culture insert (Millipore Corporation) or multi-electrode array dish (MED, Alpha MED Scientific) pre-coated with collagen (Cellmatrix type 1-C; Nitta Gelatin), as described previously^55^. Briefly, the slice was cultured in air at 36.5 °C with 1.2 ml Dulbecco’s modified Eagle’s medium (Invitrogen) with 0.1 mM D-luciferin K and 5% supplement solution. After 5–22 days, the cultured SCN slices were subjected to the continuous measurement of bioluminescence and spontaneous firing.

### Measurements of bioluminescence

Bioluminescence from the whole SCN slice was measured with a luminometer equipped with a PMT (Lumicycle, Actimetrics) as previously described^55^. The intensity of bioluminescence measured for 1 min at 10 min intervals was expressed in relative light units (RLU; counts/min).

Bioluminescence in time-series images was measured using an electron-multiplying (EM) charge-coupled device (CCD) (ImagEM; Hamamatsu Photonics) or CCD (ORCA-ІІ; Hamamatsu Photonics) camera attached to the bottom port of ECRIPSE E1000 (Nikon) and the top port of ECRIPSE TE2000-U or ECLIPSE 80i (Nikon). The images were captured every 60 min with an exposure time of 59 min. The intensity of bioluminescence was expressed in relative light units (RLU; counts/h) and analyzed at pixel level of 2.3 μm × 2.3 μm for ImagEM and 4.3 μm × 4.3 μm for ORCA-ІІ.

### Simultaneous measurements of spontaneous firing and PER2::LUC in the SCN slice

Extracellular action potential was continuously recorded for more than 5 days in the SCN slice cultured on a MED probe with 64 planar electrodes (20 × 20 µm; MED-P210A) arranged in an 8 × 8 grid embedded in the center of a 0.7 mm × 0.7 mm area (Alpha MED Scientific). Spike discharges with signal-noise ratio >2.0 were collected by Spike Detector software (Alpha MED Scientific) as previously described^55^. The number of spikes was calculated in 1 min or 100 ms bins for each electrode covered by the SCN slice. The SCN slice on a MED probe was placed in a mini-incubator installed on the stage of an upright microscope (ECRIPSE E1000, Nikon) or inverted microscope (ECRIPSE TE2000-U, Nikon) for simultaneous recording of PER2::LUC bioluminescence and spontaneous firing.

### Simultaneous measurements of PER2::LUC, spontaneous firing, and calcium levels in the SCN slice

The SCN slice was cultured on a Millicell-CM culture insert. Adeno-associated virus (AAV; serotype rh10) was harbored with GCaMP6s, a genetically encoded calcium sensor, under the control of human synapsin-1 promoter (University of Pennsylvania Gene Therapy Program Vector Core). An aliquot of AVV was inoculated onto the surface of the cultured SCN slice 3–5 days after the preparation. The next day of AAV infection, the SCN slice was transferred onto the MED probe. After culturing for 7–14 days on the MED, simultaneous measurements of bioluminescence, fluorescence, and neuronal activity were started. The MED was placed in a mini-incubator installed on the stage of an upright microscope (ECRIPSE-80i, Nikon) equipped with an EM-CCD camera (ImagEM, Hamamatsu Photonics). Fluorescence and bioluminescence were recorded with an EM-CCD camera at −80 °C every 60 min with an exposure time of 2–3 s and 59 min, respectively. For the measurement of fluorescence, an LED-based light source (Retra Light Engine; Lumencor) was used. Fluorescent calcium sensor (GCaMP6s) was excited at cyan color (475/28 nm) with LED light source, and visualized with 495 nm dichroic mirror and 520/35 nm emission filters (Semlock).

### AAV injection into the SCN of adult mice in vivo

Mice of 8–10 months old were anesthetized with isoflurane (induction dose ~2%, maintenance dose ~1%) and placed in a stereotaxic instrument (Kopf). Next, 120 nl of AAV9-hSyn-GFP-Cre (3.3 × 10^12^ GC/ml) or AAV9-hSyn-hrGFP (1.5 × 10^13^ GC/ml) was injected into the SCN of VGAT^flox/flox^ mice bilaterally (±0.0 mm posterior, ±0.2 mm lateral, −5.7 mm ventral from Bregma) with a glass micropipette and an air pressure injection system (BJ-110, BEX).

### Measurement of behavioral activity

Spontaneous movements were measured by a passive infrared sensor which detects changes in animal thermal radiation due to movement^73^. The amount of movement was recorded every minute with computer software (Clocklab, Actimetrics).

### Immunohistochemical studies

After recording locomotor activity, mice were anesthetized with pentobarbital (50 mg/kg, i.p.) and perfused with saline followed by a 10% formalin solution (Wako). Brains were extracted and post-fixed in the same solution for 24 h at 4 °C, followed by a 30% sucrose solution at 4 °C for at least 2 days. 40 μm coronal sections of the SCN were made using a cryostat (Leica) and stored at 4 °C in PBS. Immunostaining was performed as previously described^55^. The primary antibody was rabbit anti-VGAT (1:1000 dilution, SIGMA) and the secondary was CF 594-conjugated Donkey anti-rabbit antibody (1:1000 dilution, Biotium). Brain sections were counterstained with DAPI and mounted to examine with a fluorescence microscope (BZ-9000, Keyence). Fluorescent images were captured with the same intensity and expose time. The intensity of VGAT was analyzed with the ImageJ software. Every third slice (in total, 4 slices) was collected from each brain for analysis. The border of the SCN was determined with the aid of DAPI-stained images. The average intensity of VGAT staining was calculated for the four slices and expressed as intensity/pixel.

### Data analysis

The properties of circadian rhythm in bioluminescence signals were analyzed at pixel level, using a cosine curve fitting method as described previously^74,75^. Rayleigh plot was also used for analyses with Oriana4 (Kovach Computing Services). The circadian bioluminescence rhythm recorded with a PMT was evaluated by a Chi-square periodogram (ClockLab, Actimetrics) using records of 7 consecutive days with a significance level of *p* < 0.01. Circadian amplitude was calculated by subtracting the circadian trough value from the peak in a cycle and was further standardized by dividing this amplitude by the circadian peak value to eliminate possible slice biases as described previously^55^. A correlation coefficient of spontaneous firing was calculated by Matlab (Mathworks). Circadian period of behavioral rhythm was determined by a Chi-square periodogram using records of 28 consecutive days under DD. The onset of behavioral rhythms was determined with the Clocklab software. When the GFP signals were not observed in the SCN with post-hoc immunochemistry, all data of the corresponding animal (one of nine VGAT^flox/flox^ mice) were excluded from the analyses. Cre-GFP was observed in the nucleus while hrGFP (control) was expressed in the cell body and axon; GFP signals in control mice were often detected outside the SCN as well.

### Statistics

Student’s *t*-test was used when two independent group means were compared, and Welch’s *t*-test was used when the variances of two group means were different. Paired *t*-test was used when two dependent group means were compared. A one-way ANOVA with a post-hoc Tukey–Kramer test was used to analyze a single time series data. A two-way ANOVA with post-hoc *t*-test was adopted when two independent time series data were compared (Statview or Statcel 3).

### Reporting summary

Further information on research design is available in the Nature Research Reporting Summary linked to this article.

## Supplementary information

Description of Additional Supplementary Files

Supplementary Information

Reporting Summary

Supplementary movie 1

Supplementary movie 2

Supplementary movie 3

Supplementary movie 4

## Data Availability

The data that support the findings of this study are available from the corresponding author on reasonable request.
