# Supplementary material for: GABA in the suprachiasmatic nucleus refines circadian output rhythms in mice
Source: Commun Biol. 2019 Jun 21;2:232. doi: 10.1038/s42003-019-0483-6 (PMC6588595; doi:10.1038/s42003-019-0483-6)
Supplement: Supplementary file 1 — Description of Additional Supplementary Files [file 42003_2019_483_MOESM1_ESM.docx]

Description of additional supplementary items

**Supplementary movie1**: **Representative video image of circadian PER2::LUC and GCaMP6s rhythms in the WT SCN.**

Fluorescence (right) and bioluminescence (left) in the same WT SCN were recorded with an EM-CCD camera at 1 hour intervals with an exposure time of 2-3 sec and 59 min, respectively.

**Supplementary movie 2: Representative video image of circadian PER2::LUC and GCaMP6s rhythms in the VGAT^-/-^ SCN.**

Fluorescence (right) and bioluminescence (left) in the same VGAT^-/-^ SCN were recorded with an EM-CCD camera at 1 hour intervals with an exposure time of 2-3 sec and 59 min, respectively.

**Supplementary movie 3: Representative video image of Ca^2+^ imaging in the WT and VGAT^-/-^ SCN.**

Fluorescence signals in the WT (left) and VGAT^-/-^ (right) SCN were recorded with an EM-CCD camera with an exposure time of 330 msec at 3 sec intervals for 30 min. Calcium spikes were observed frequently in the VGAT^-/-^ SCN.

**Supplementary movie 4: Representative video image of Ca^2+^ imaging with a higher time resolution in the WT and VGAT^-/-^ SCN.**

Fluorescence signals in the WT (left) and VGAT^-/-^ (right) SCN were recorded with an EM-CCD camera with an exposure time of 50 msec at 100 msec intervals for 1 min. Calcium spikes were observed once at around 20,000 msec in the VGAT^-/-^ SCN.
