# Supplementary material for: GABA in the suprachiasmatic nucleus refines circadian output rhythms in mice
Source: Commun Biol. 2019 Jun 21;2:232. doi: 10.1038/s42003-019-0483-6 (PMC6588595; doi:10.1038/s42003-019-0483-6)
Supplement: Supplementary file 2 — Supplementary Information [file 42003_2019_483_MOESM2_ESM.pdf]

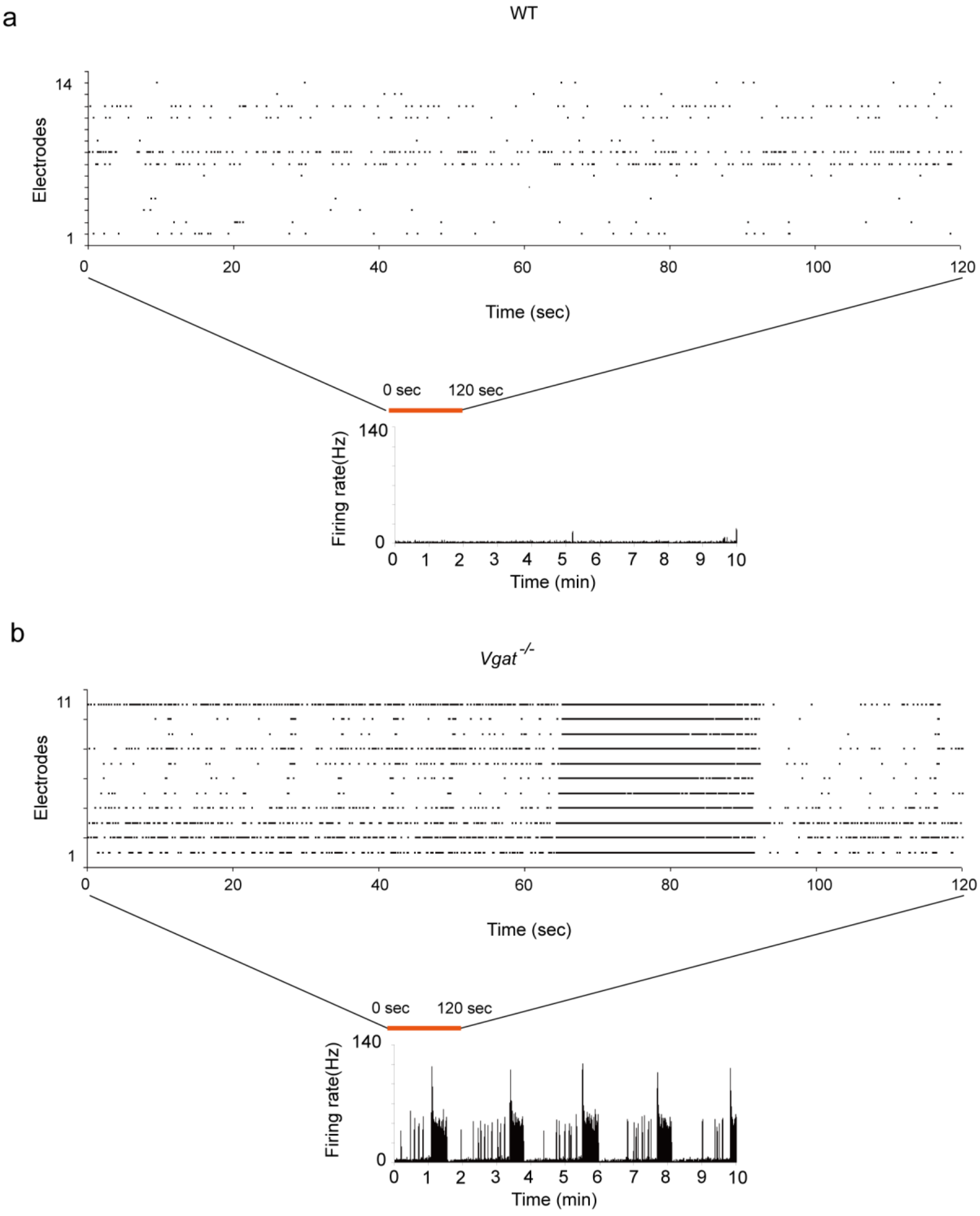


**Supplementary figure 1: Time stamp of spontaneous firing and mean firing patterns in the fetal SCN slice on MED**

Spontaneous firings expressed with black dots from each electrode for 120 sec (upper) at CT12 and the mean firing rate (Hz) of all electrodes located on the SCN slice (lower) of the WT (a) and VGAT$^{-/-}$ (b) mouse. The number of electrodes in the SCN region was 14 in WT and 11 in VGAT$^{-/-}$ slices.

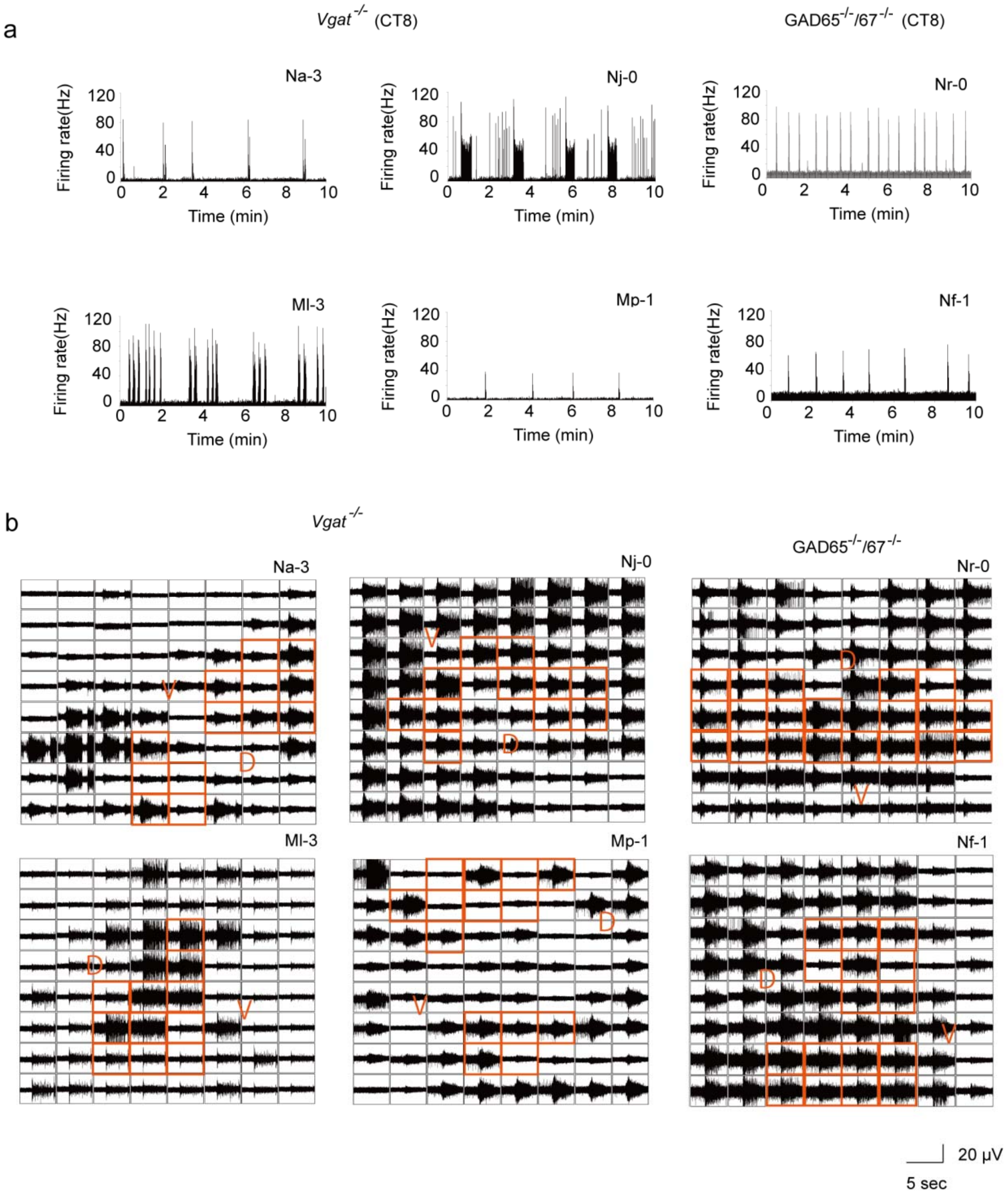


**Supplementary figure 2: Various burst firings in the VGAT$^{-/-}$ fetal SCN**

(a) Mean firing rates (Hz) in 100 msec bins for 10 min at CT8 in four VGAT$^{-/-}$ and two GAD65$^{-/-}$/67$^{-/-}$ SCN slices. A sign at the upper right corner indicates the slice number. (b) Extracellular recording of neuronal activity for 5 sec from 64 electrodes located on a SCN slice from VGAT$^{-/-}$ and GAD65$^{-/-}$/67$^{-/-}$ mice. Red squares superimposed on the record indicate the areas covered by the SCN slice and red letters the regions of SCN (V, ventral; D, dorsal)

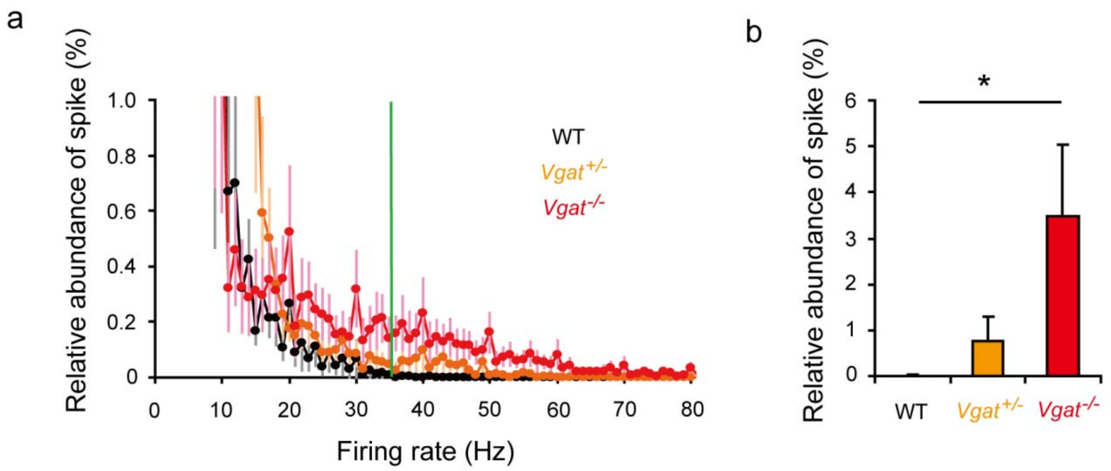


**Supplementary figure 3: Distribution of burst firing abundance in fetal VGAT$^{-/-}$ SCN slices**

(a) Firing rate (Hz) was calculated in 100msec bins at CT8 and its distribution was expressed in % of the total bins (6,000) in WT (black, n = 5), VGAT$^{+/-}$ (orange, n = 4) and VGAT$^{-/-}$ (red, n = 6) SCN slices. The WT SCN did not exhibit firing rate higher than 35 Hz (indicated by the green line). In the VGAT$^{-/-}$ SCN, firing rate exceeded 35 Hz in substantial number of bins, while this was observed only in a small number in the VGAT$^{+/-}$ SCN. (b) The number of bins exceeding 35 Hz (burst firing) was calculated for each genotype and expressed as % of the total bins. The ratio of bins that showed burst firing was significantly higher in the VGAT$^{-/-}$ SCN than in the WT SCN (*; $P < 0.05$, Kruskal-Wallis with a post-hoc Steel-Dwass's test). Data are expressed as a mean and SD.

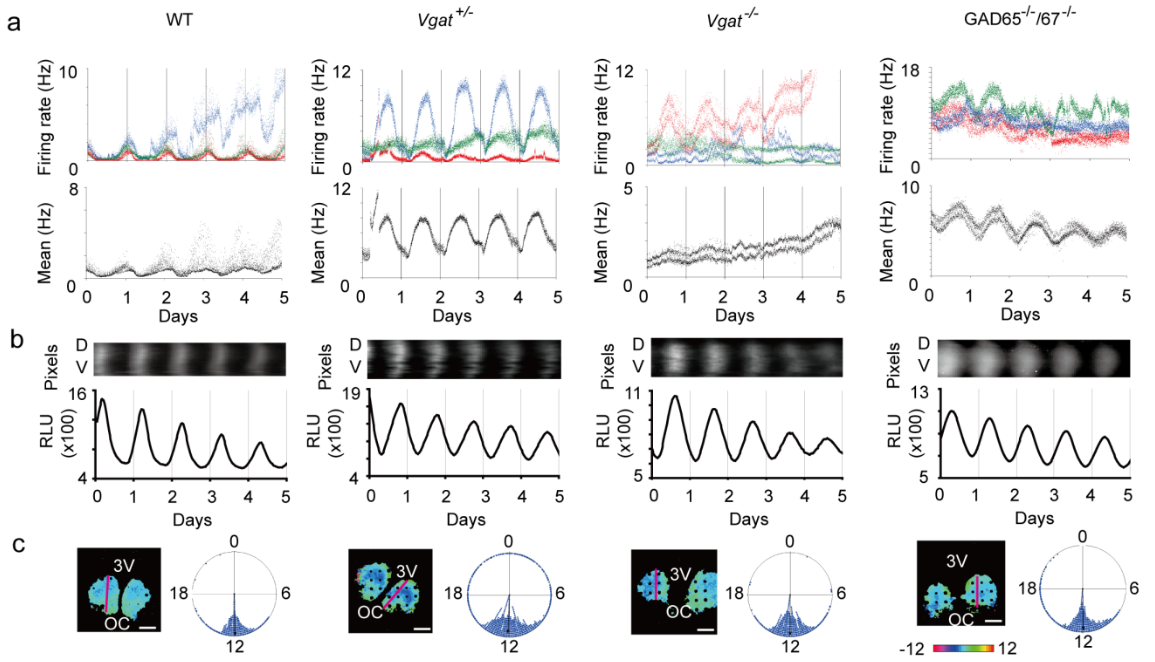


**Supplementary figure 4: Circadian firing and PER2::LUC rhythms in the $VGAT^{-/-}$ and $GAD65^{-/-}/67^{-/-}$ fetal SCN**

(a) Spontaneous firing rate (Hz) plotted in 1 min bins for 5 circadian cycles from three electrodes (upper) and the averaged firing rate of all electrodes (n = 15, 14, 13, and 19 for WT, $VGAT^{+/-}$, $VGAT^{-/-}$ and $GAD65^{-/-}/67^{-/-}$) (lower) located on the SCN region of WT (far left), $VGAT^{+/-}$ (left), $VGAT^{-/-}$ (right), and $GAD65^{-/-}/67^{-/-}$ (far right) mice. The different colors indicate the firing rhythms from different electrodes of an MED. (b) Circadian rhythms of PER2::LUC at pixel level illustrated in a line scan (1 pixel width) from dorsal (D) to ventral (V) SCN (upper) and on whole tissue level (lower). The site of line scan is shown as a red line in the corresponding acrophase map above. (c) An acrophase map of PER2::LUC rhythms (left) and a distribution of acrophase of each pixel in a form of Rayleigh plot (right). Black spots in the phase map indicate electrodes. Scale bars represent 200 μm. OC: optic chiasm, 3V: third ventricle

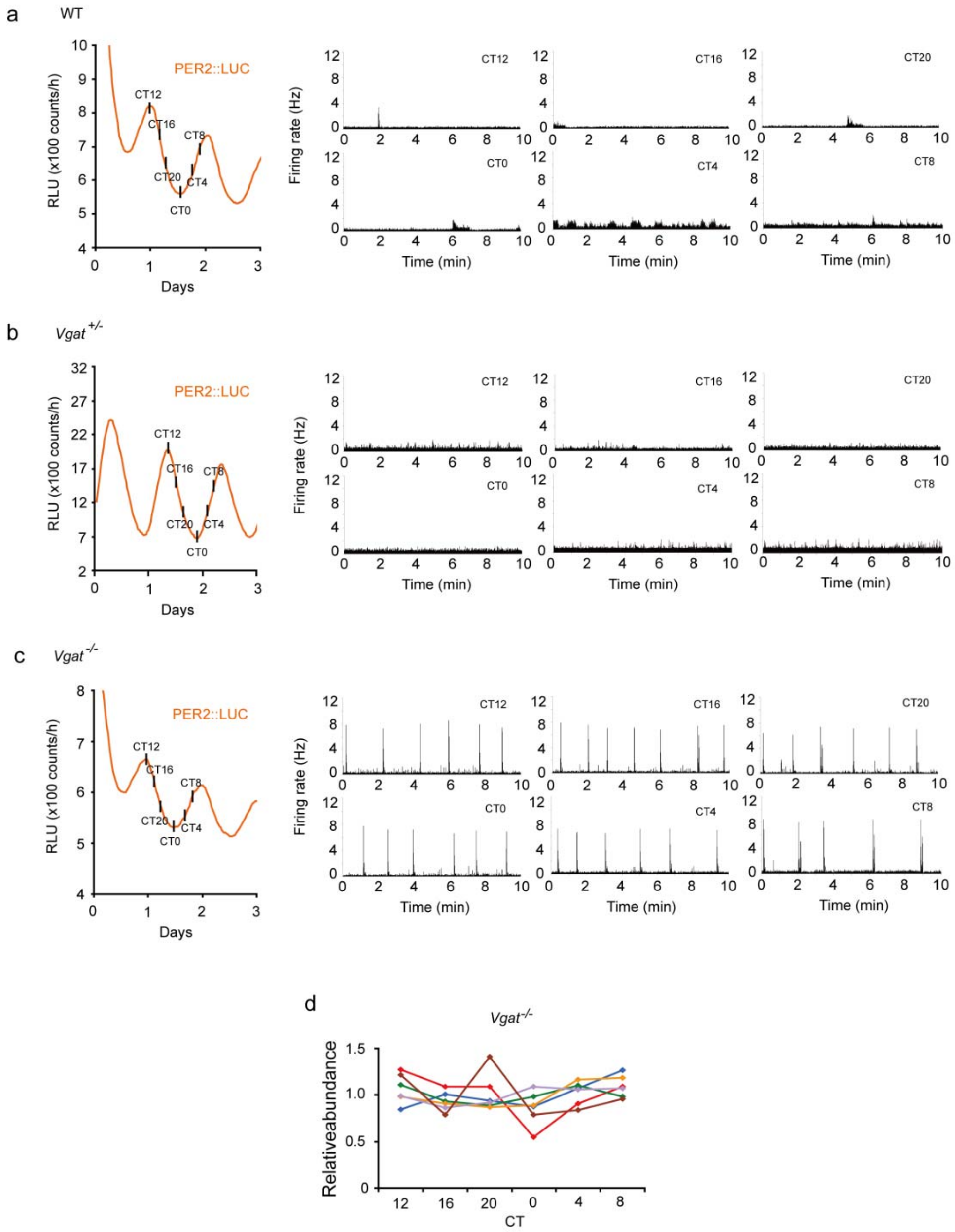


**Supplementary figure 5: Circadian phase independence of burst firings in the VGAT$^{-/-}$ fetal SCN.**

(a, b, c) Mean frequency (Hz) of spontaneous firing at CT12, 18, 20, 0, 4 and 8 in a single WT (A), VGAT$^{+/-}$ (b), and VGAT$^{-/-}$ (c) SCN slice on MED (left). Simultaneously determined PER2::LUC rhythms were also illustrated with CT indications (right). (d) Relative abundance of burst firing (>35 Hz) expressed as % of individual mean is plotted against the time of day (CT) in the VGAT$^{-/-}$ SCN. Different colors indicate the different SCN slices (n = 6). Significant difference was not detected in abundance during the circadian cycle.

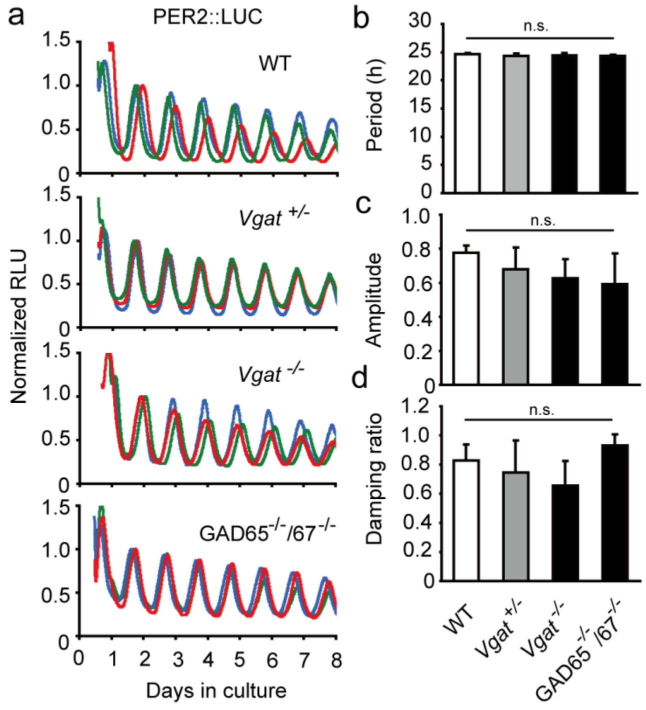


**Supplementary figure 6: Circadian PER2::LUC rhythms in the SCNs of four different genotypes**

(a) Four representative PER2::LUC rhythms for the WT (uppermost, n = 5), VGAT$^{+/-}$ (upper, n = 14), VGAT$^{-/-}$ (lower, n = 9) and GAD65$^{-/-}$/67$^{-/-}$ (lowermost, n= 4) SCN. Different colors represent different SCN slices. The average and standard deviation of the circadian period calculated by chi-square periodogram (b), standardized amplitude (c) and damping ratio (the ratio of amplitude on the first and the sixth cycle). n.s.: not significant (period, $P$ = 0.458; Amplitude, $P$ = 0.105; Damping ratio, $P$ = 0.0857, one-way ANOVA)

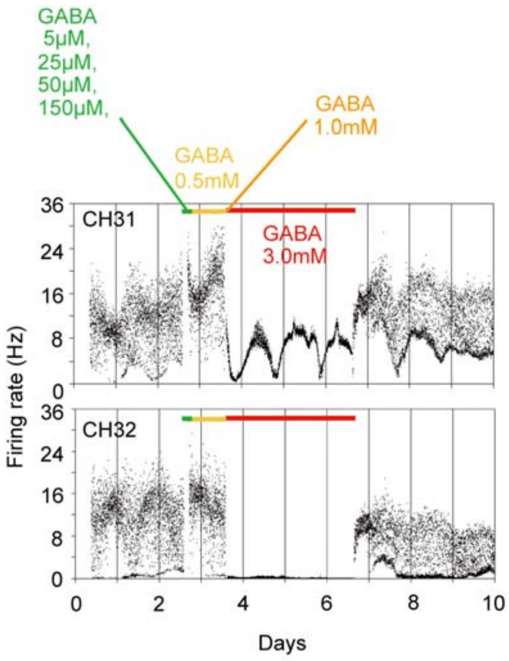


**Supplementary figure 7: Effects of GABA on burst firings in the VGAT$^{-/-}$ fetal SCN slice**

Effects of GABA on circadian firing rhythms measured at 1 min bins in the VGAT$^{-/-}$ SCN slice on MED. GABA of 5 to 150 μM was applied at the time indicated by the green bar, and then 0.5 mM GABA was applied for 1 day (yellow bar). At the point indicated by an orange bar, 1.0 mM GABA was applied, which was immediately followed by 3.0 mM GABA application for 3 days (red bar). Burst firings were suppressed but the circadian rhythm persisted with 3.0 mM GABA. CH31, CH32 denote the number of electrodes.

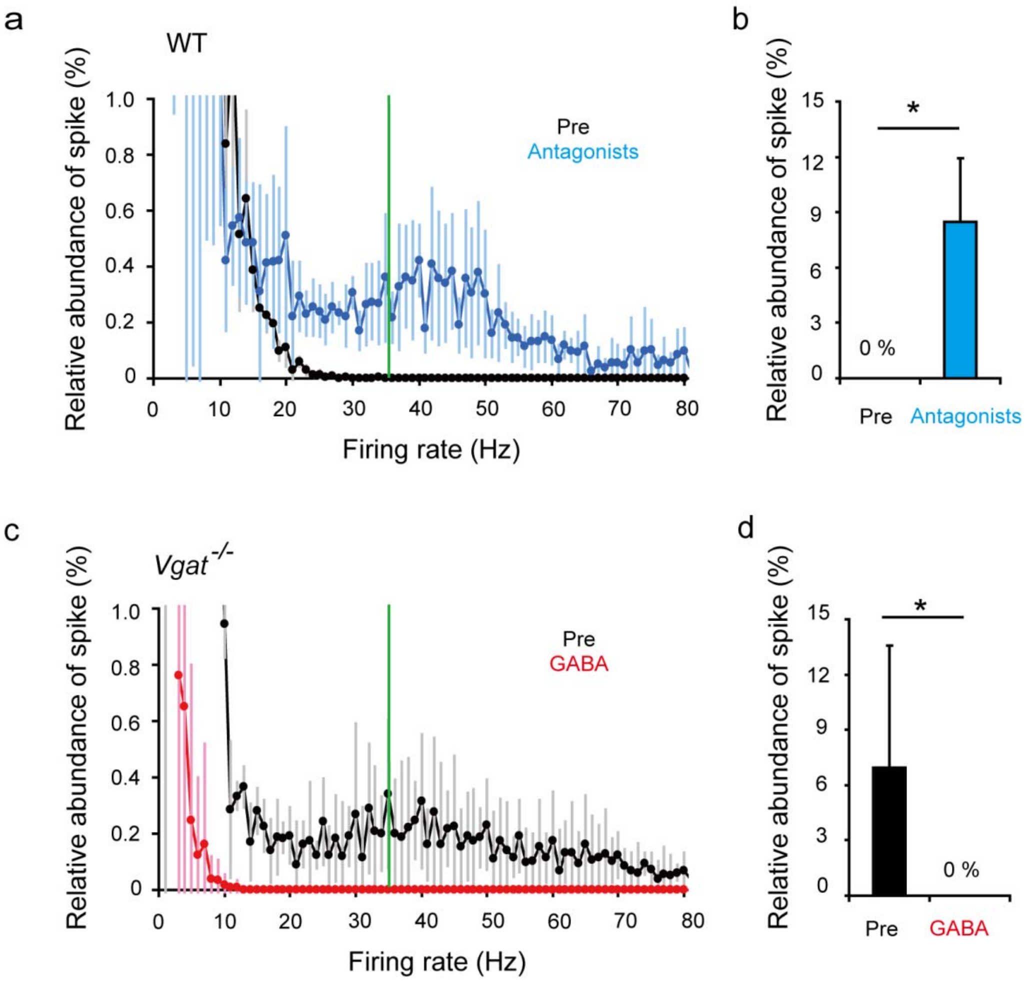


**Supplementary figure 8: Effects of GABA antagonist or GABA on the distribution of firing rate in the WT and VGAT$^{-/-}$ fetal SCN**

(a) The distribution of the firing rate in 100 msec bins was expressed as % of 10 min measurement (6,000 bins) in the WT (upper) and VGAT$^{-/-}$ (lower) SCN slice. See also the legend of figure S3. A substantial number of bins exceeded 35 Hz (green vertical line) in the WT SCN after application of GABA antagonists. (b) The number of bins exceeding 35 Hz was significantly increased after application of GABA antagonists (blue) compared to the pre-application level (black). (c) A firing rate of a larger frequency than 10Hz was not detected during GABA application in the VGAT$^{-/-}$SCN slice (red) as compared to the pre-application level (black). (d) The number of bins exceeding 35 Hz was significantly decreased after application of GABA (*; $P$ < 0.05, Kruskal-Wallis with a post-hoc Steel-Dwass's test).
