# Supplementary material for: GABA in the suprachiasmatic nucleus refines circadian output rhythms in mice
Source: Commun Biol. 2019 Jun 21;2:232. doi: 10.1038/s42003-019-0483-6 (PMC6588595; doi:10.1038/s42003-019-0483-6)
Supplement: Supplementary file 3 — Reporting Summary [file 42003_2019_483_MOESM3_ESM.pdf]

nature research

Corresponding author(s): Daisuke Ono and Sato Honma

Last updated by author(s): Dec 17, 2018

Reporting Summary

Nature Research wishes to improve the reproducibility of the work that we publish. This form provides structure for consistency and transparency in reporting. For further information on Nature Research policies, see Authors & Referees and the Editorial Policy Checklist.

Statistics

For all statistical analyses, confirm that the following items are present in the figure legend, table legend, main text, or Methods section.

n/a	Confirmed	
☐	☒	The exact sample size (*n*) for each experimental group/condition, given as a discrete number and unit of measurement
☐	☒	A statement on whether measurements were taken from distinct samples or whether the same sample was measured repeatedly
☐	☒	The statistical test(s) used AND whether they are one- or two-sided<br>*Only common tests should be described solely by name; describe more complex techniques in the Methods section.*
☐	☒	A description of all covariates tested
☐	☒	A description of any assumptions or corrections, such as tests of normality and adjustment for multiple comparisons
☐	☒	A full description of the statistical parameters including central tendency (e.g. means) or other basic estimates (e.g. regression coefficient) AND variation (e.g. standard deviation) or associated estimates of uncertainty (e.g. confidence intervals)
☐	☒	For null hypothesis testing, the test statistic (e.g. *F*, *t*, *r*) with confidence intervals, effect sizes, degrees of freedom and *P* value noted<br>*Give P values as exact values whenever suitable.*
☒	☐	For Bayesian analysis, information on the choice of priors and Markov chain Monte Carlo settings
☐	☒	For hierarchical and complex designs, identification of the appropriate level for tests and full reporting of outcomes
☒	☐	Estimates of effect sizes (e.g. Cohen's *d*, Pearson's *r*), indicating how they were calculated

Our web collection on statistics for biologists contains articles on many of the points above.

Software and code

Policy information about availability of computer code

Data collection	HC-image (Hamamatsu photonics) for acquiring of imaging of the cultured SCN slice. Llumicycle (actimetrics) for recording of bioluminescence from the cultured SCN slice. Spike Detector (Alpha MED Scientific) for recording of spontaneous firings in the cultured SCN slice. ClockLab (actimetrics) for recording of behavioral rhythms of mice.
Data analysis	ClockLab (actimetrics) for analysis of circadian rhythms of bioluminescence of the cultured SCN slice and behavioral rhythms of mice. Oriana4 (Kovach Computing Services) for analysis of circadian phase of imaging data. ImageJ, microsoft Excel, and Matalab were also used.

For manuscripts utilizing custom algorithms or software that are central to the research but not yet described in published literature, software must be made available to editors/reviewers. We strongly encourage code deposition in a community repository (e.g. GitHub). See the Nature Research guidelines for submitting code & software for further information.

Data

Policy information about availability of data

All manuscripts must include a data availability statement. This statement should provide the following information, where applicable:
- Accession codes, unique identifiers, or web links for publicly available datasets
- A list of figures that have associated raw data
- A description of any restrictions on data availability

All the data are available from corresponding author upon suitable request .

Field-specific reporting

Please select the one below that is the best fit for your research. If you are not sure, read the appropriate sections before making your selection.

☒ Life sciences ☐ Behavioural & social sciences ☐ Ecological, evolutionary & environmental sciences

For a reference copy of the document with all sections, see nature.com/documents/nr-reporting-summary-flat.pdf

Life sciences study design

All studies must disclose on these points even when the disclosure is negative.

Sample size	Sample sizes for behavioral, histochemical, imaging, and electrophysiological studies were based on previous experiments from our laboratory and others, which had been demonstrated to be capable of detecting significant changes.
Data exclusions	No data were excluded.
Replication	Experiments were performed with sufficient animals per group to demonstrate statistical significance.
Randomization	Littermate was used for the experiments.
Blinding	The investigators were not blinded to group allocation during data collection and/or analysis, but we objectively recorded and analyzed data.

Reporting for specific materials, systems and methods

We require information from authors about some types of materials, experimental systems and methods used in many studies. Here, indicate whether each material, system or method listed is relevant to your study. If you are not sure if a list item applies to your research, read the appropriate section before selecting a response.

Materials & experimental systems

n/a	Involved in the study	
☐	☒	Antibodies
☒	☐	Eukaryotic cell lines
☒	☐	Palaeontology
☐	☒	Animals and other organisms
☒	☐	Human research participants
☒	☐	Clinical data

Methods

n/a	Involved in the study	
☒	☐	ChIP-seq
☒	☐	Flow cytometry
☒	☐	MRI-based neuroimaging

Antibodies

Antibodies used	rabbit anti-VGAT (sigma, SAB2700790)
Validation	Validation: Primary rabbit anti-VGAT (sigma, SAB2700790) was used at a dilution of 1:1000. The antibody has been used for immunohistochemistry previously (Husson et. al., 2014, J. Neurosci; White et. al., 2014, J. Neurosci.; Soykan et. al., 2014, EMBO J.). All secondary antibodies used in this study have been commercial validated and widely used/validated by the field.

Animals and other organisms

Policy information about studies involving animals; ARRIVE guidelines recommended for reporting animal research

Laboratory animals	VGAT-/- and GAD65-/-/67-/- mice of C57BL/6J background and Vgatflox/flox mice of 129/SvEv (back crossed with C57BL/6J mice at least two generations) were crossed with mPer2Luc knock-in mice carrying a PER2::LUC fusion reporter. Both male and female mice were used. For slice culture, mice were used at embryonic day 19 or 20. For AAV injection experiment, we started recording of behavior of mice at 7-10 weeks old.
Wild animals	n.a.
Field-collected samples	n.a.
Ethics oversight	Experiments were conducted in compliance with the rules and regulations established by the Animal Care and Use Committee of Hokkaido University under the ethical permission of the Animal Research Committee of Hokkaido University (Approval No.08-0279), that of Gunma University (Approval No.07-109, No.09-047, No.14-006), and that of Nagoya University (Approval NO. 18257).

Note that full information on the approval of the study protocol must also be provided in the manuscript.
